# Unexpected scaffold rearrangement product of pirenzepine found in commercial samples

**DOI:** 10.1038/s41598-021-02732-y

**Published:** 2021-12-03

**Authors:** Marius Ozenil, Lukas Skos, Alexander Roller, Natalie Gajic, Wolfgang Holzer, Helmut Spreitzer, Sonja Platzer-Ozenil, Chrysoula Vraka, Marcus Hacker, Wolfgang Wadsak, Verena Pichler

**Affiliations:** 1 Division of Nuclear Medicine, Department of Biomedical Imaging and Image-Guided Therapy, Medical University of Vienna, Vienna, Austria; 2 X-Ray Structure Analysis Centre, Faculty of Chemistry, University of Vienna, Vienna, Austria; 3 Department of Pharmaceutical Sciences, Faculty of Life Sciences, University of Vienna, Vienna, Austria; 4 Faculty of Chemistry, University of Vienna, Vienna, Austria; 5 CBmed GmbH - Center for Biomarker Research in Medicine, Graz, Austria

**Keywords:** Drug safety, Chemical safety

## Abstract

Pharmacovigilance aims at a better understanding of the molecular events triggered by medications to prevent adverse effects, which despite significant advances in our analytical repertoire plague the use of drugs until today. In this study, we find that clinically prescribed and commercially available pirenzepine may not be the correct compound. Pirenzepine can undergo an unexpected scaffold rearrangement from the pharmaceutical active ingredient (API) to a previously uncharacterized benzimidazole. The rearrangement occurs under highly acidic conditions, which were believed to favour the dihydrochloride formation of pirenzepine. The rearranged products of pirenzepine and the structurally related telenzepine have significantly decreased affinity for the muscarinic acetylcholine receptor, the pharmacological target of these compounds. Fortunately, in situ rearrangement after oral application is no safety issue, as we show that reaction kinetics in gastric acid prevent rearrangement. The research community should consider appropriate measures to perform reliable receiving inspections in the commercial supply of well described and frequently used chemicals, in particular if experiments yield unexpected results.

## Introduction

The chemical industry is currently being reshaped by globalization and several producers in emerging markets have become global leaders recently^1^. Consequently, the market of laboratory chemicals is characterized by a boom of chemical distributors^2^, which import bulk quantities from companies around the world to be sold in small quantities to research institutions. As active pharmaceutical ingredients (APIs) are chemicals by their very nature, reliable quality control of the chemical identity is the basis of drug safety. The pharmaceutical industry is responsible for at least hundreds of deadly incidents due to confusion of chemicals in their products during the last 30 years. For example, Baxter processed heparin containing oversulfated chondroitin sulfate from a commercial supplier in 2008^3^ and instead of glycerin falsely specified diethylene glycol has been used in the production of cough syrup between 1992 and 2007^4^.

Pirenzepine is a well-established parasympatholytic drug, which is in clinical use since the late 1970s^5^. It acts as antagonist for the muscarinic acetylcholine receptors (mAChRs), with a strong preference for the mAChR subtype M1. The drug triggers only peripheral mAChRs, as it fails to cross the blood brain barrier. Pirenzepine is best known for its long history as inhibitor of gastric acid secretion and gastric motility in the treatment of gastric and duodenal ulcers^6^. Besides these classical applications, pirenzepine is effective for controlling myopia progression in children^7^ and is currently undergoing a phase II clinical trial for the treatment of peripheral neuropathy^8^. The relevance of pirenzepine roots in the early days of mAChR research, when different binding affinities provided evidence for the existence of several mAChR subtypes^9^. Since the compound is widely used in biological assays to differentiate between M1 and the other mAChR subtypes^10,11^, our knowledge about these receptors is tightly linked to the chemical structure of pirenzepine.

**Figure 1:**
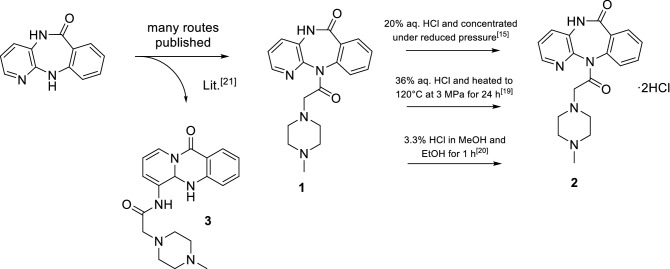
Overview of synthetic routes of pirenzepine dihydrochloride. Emphasis is given on the synthetic conditions for the formation of the dihydrochloride.

Pirenzepine (**1**) is mainly distributed as dihydrochloride (**2**) and the active pharmaceutical ingredient is widely available in more than 25 marketed products^12–14^. The first synthesis of pirenzepine dihydrochloride was patented by Dr Thomae GmbH^15^ and many alternative synthesis procedures were reported since (Fig. 1)^16–20^. Budesinsky et al*.*^21^ reported an unexpected side product (**3**) formed during the synthesis of **1**, in which the 5,11-dihydro-6*H*-pyrido-[2,3-*b*][1,4]benzodiazepin-6-one part rearranges to 6-amino-5,5*a*-dihydro-11*H*-pyrido[2,1-*b*]quinazolin-11-one. Generally, the well-studied class of β-lactam antibiotics illustrates that lactams can be prone to hydrolysis^22^ and rearrangement^23^, especially when under high ring strain. Despite intensive investigations on the synthesis of pirenzepine, only few reports provide experimental details of the final step, the reaction of pirenzepine to its dihydrochloride (Fig. 1)^15,19,20^. Furthermore, pirenzepine acts as a lead structure for the development of novel mAChR ligands. The most notable examples which arose from this strategy are the mAChR M1 antagonist telenzepine^24^, the M2/M4 antagonist AF-DX-384^25^ and the M2 antagonists AF-DX 116^26^ and AQ-RA 741^27^.

We obtained pirenzepine dihydrochloride from a fine chemical distributor for displacement studies on mAChR M1^28^. However, our experiments indicated no pirenzepine dihydrochloride was present in our sample, which was later determined to contain a structural isomer. We went on to characterize this structural isomer, study its mAChR affinity profile and determine the rearrangement reaction leading to its formation. mAChR research uses in addition to pirenzepine the structural relative telenzepine, which we found to undergo a similar rearrangement. The rearranged products (**4** and **5**) were fully characterized by NMR, HRMS, X-ray and HPLC as well as their affinity determined towards all subtypes of mAChRs. Finally, we would like to note that the previously unrecognized rearrangements of pirenzepine and analogues offer a useful tool for the synthesis of other *N*-substituted imidazoles.

## Results and discussion

### Chemistry

**Figure 2:**
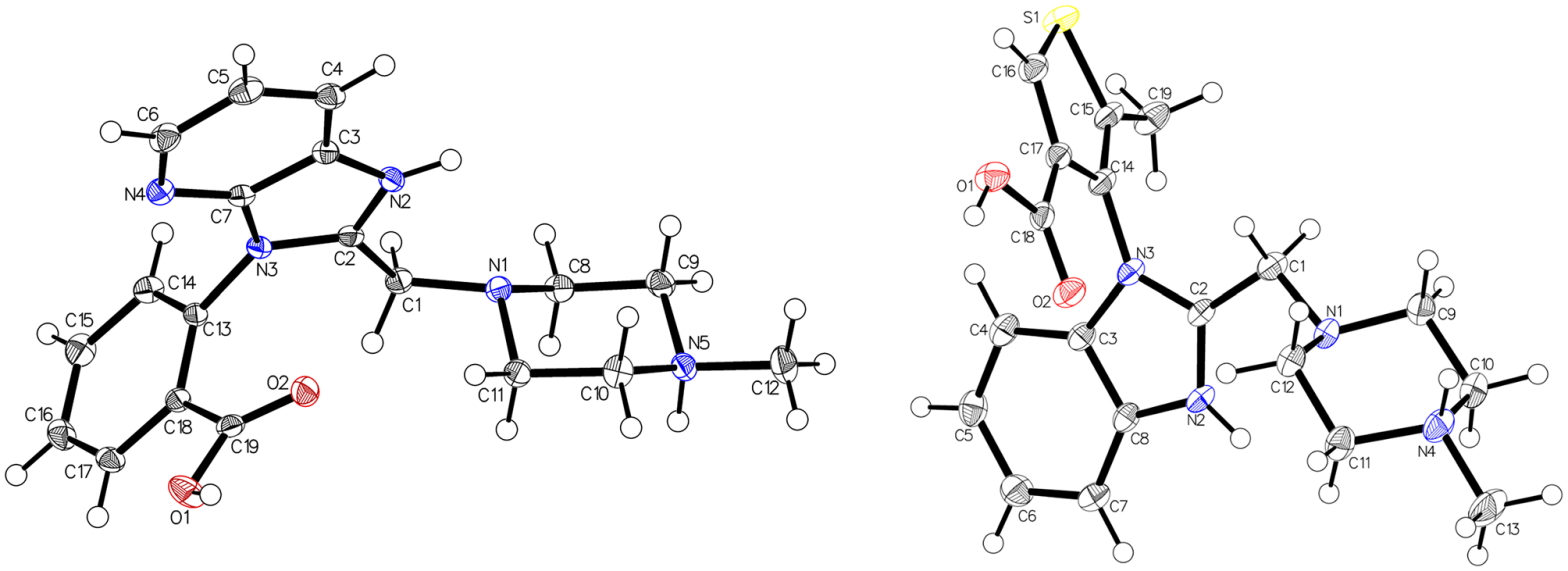
Crystal structure of **4** (left) and **5** (right), drawn with 50% displacement ellipsoid. The bond precision for C–C single bonds is 0.0017 Å (**4**) and 0.0039 Å (**5**), respectively (Supplementary Tables S5–S8). Solvent omitted for clarity.

**Figure 3:**
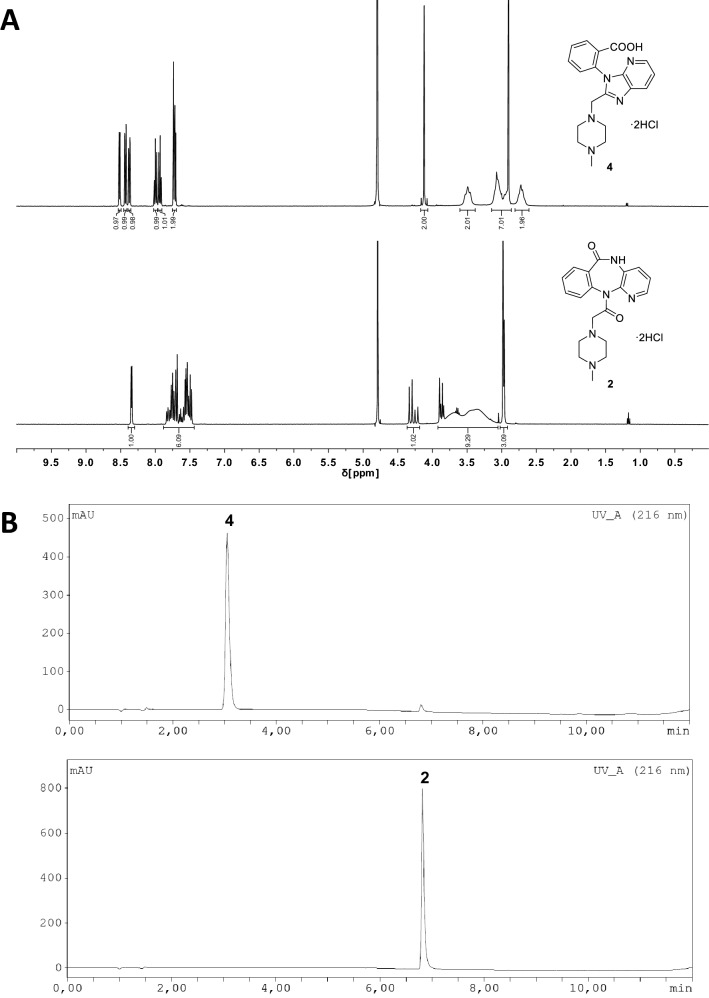
A: ^1^H-NMR spectra of **4** (top, sample #2) and **2** (bottom, sample #4) in D_2_O at 400 MHz. B: UV-HPLC traces of **4** (top, sample #2) and **2** (bottom, sample #4).

X-ray measurements revealed the true structure (Fig. 2) of a commercially obtained compound, which did not contain the anticipated pirenzepine dihydrochloride (**2**), but a constitutional isomer (**4**) not yet reported in the literature. Isomers **4** and **2** cannot be distinguished by mass spectrometry without molecular fragmentation. However, ^1^H-, ^13^C-, ^15^ N-NMR spectroscopy, IR spectroscopy, melting point and RP-HPLC allow for a clear differentiation (Fig. 3 and Supplementary Figs. S1, S2, Table S3).

**Figure 4:**
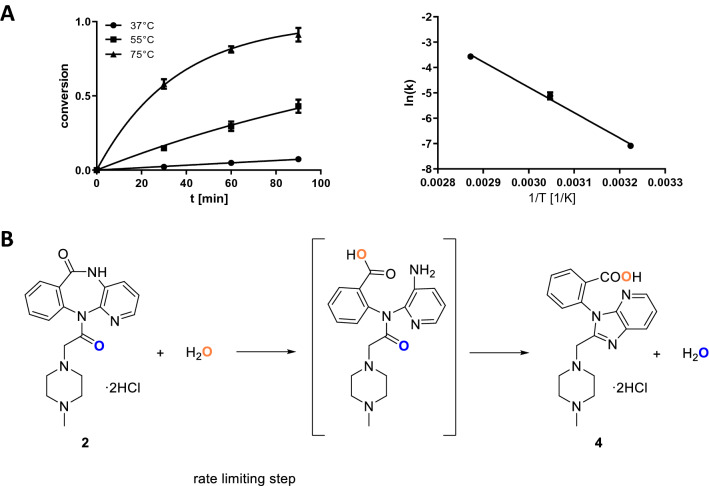
A: Kinetic data of the rearrangement of **2** in 6 M HCl at an initial concentration of 50 µg/mL as determined by HPLC. Error bars indicate the standard deviation of n = 3 measurements. Left: The kinetic profile of the rearrangement shows the conversion of **2** to **4** as function of time at different temperatures. The conversion is calculated normalized to the maximum area under the curve of **4**. The rearrangement follows a pseudo first order kinetic with half lifes of 25 ± 2 min (75 °C), 117 ± 16 min (55 °C) and 830 ± 30 min (37 °C). Right: Arrhenius plot illustrating a linear relationship between inverse temperature and logarithm of the rate constants. The activation energy of the reaction was delineated from the slope: 83.2 ± 1.9 kJ/mol. B: Reaction scheme showing the proposed reactive intermediate. Oxygens in the carboxylic acid group are indistinguisable, but for reasons of clarity only one of them is coloured like the hydrolyzing water.

In search for a synthetic access to **4** we revealed that aqueous acidic conditions combined with moderately increased temperature result in rearrangement of **2** to **4**. When **2** is reacted in 0.1 M aqueous HCl at 37 °C, 55 °C or 75 °C overnight, **4** is only formed in traces (< 1%). However, in strong excess of aqueous HCl (6 M) the rearrangement was shown to follow a pseudo first order kinetic with a quantitative conversion (Fig. 4A). We propose that this reaction proceeds via lactam hydrolysis and subsequent condensation of the resulting primary amine with the exocyclic carbonyl group (Fig. 4B). The intermediate (in brackets) was never observed experimentally, which underlines its reactiveness and indicates, that the prior amide hydrolysis is the rate limiting step. This is further supported by the observed high activation energy (83.2 ± 1.9 kJ/mol), which is common for acidic amide bond cleavages (78.6–89.9 kJ/mol)^29^. Based on the minimized MMFF94 energies of **4** (583 kJ/mol) and **2** (762 kJ/mol), the reaction of the formed intermediate towards **4** appears also thermodynamically favored as opposed to the back reaction. The exchange of an oxygen atom with a heavy oxygen by utilization of [^18^O]H_2_O during the rearrangement reaction supports the proposed reaction scheme (Fig. 8).

Given that pirenzepine is used as oral drug and that the rearrangement takes place in acidic aqueous solution, we investigated whether isomerization of **2** to **4** can occur in the gastric environment. Fortunately, neither simulated gastric fluid (0.08 M aq. HCl, 1 g/L NaCl and 1.6 g/L pepsin) nor equimolar aqueous HCl solution caused detectable isomerization of **2** to **4** after 6 h at 37 °C. These results indicate that pirenzepine formation of pharmacological relevant doses of **4** in the stomach are unlikely, leaving its formation a sole problem of chemical production and distribution. Pure, crystalline **2** heated to 100 °C for 12 h did not result in formation of **4** in detectable amounts, indicating that isomerization as a result of improper handling during delivery is not to be expected.

Conditions under which pirenzepine undergoes this rearrangement are typical conditions for the formation of hydrochlorides. Review of the chemical literature showed that in the patent of Suzhou Homesun Pharmaceutical Co., Ltd^19^ even harsher conditions (36% HCl, 120 °C, 3 MPa) are used for the dihydrochloride formation of pirenzepine. Manufacturers following this procedure will inevitably and unintentionally produce **4** instead of **2** in quantitative yields. To avoid formation of **4** during the production of **2** we propose milder conditions must be chosen^15,20^. Considering that many amines are therapeutically applied as hydrochlorides, the rearrangement of pirenzepine may not be an isolated case.

Subsequently, we obtained pirenzepine dihydrochloride batches from several distributors of fine chemicals (Supplementary Table S1) and pharmaceuticals (Supplementary Table S2) and used UV-HPLC for qualitative analysis. This revealed two affected chemical distributors (sample #2 and #3). Frequently, certificates of analysis provided by distributors contain only superficial parameters like appearance, assay and melting point. However, no substantive analytical data allowing a solid proof of the chemical structure are presented. The main pillar of quality control for assay determination is HPLC, but we should question this overreliance on a separation method that cannot provide structural information. Finally, in close collaboration with the distributors the chemical was removed from their product portfolio.

All tested pharmaceutical products containing pirenzepine were shown to contain the expected constitutional isomer (**2**). It would be of great interest to analyze pirenzepine tablets originating from Homesun Pharmaceutical Co., Ltd because this company is holding the patent for the problematic synthesis procedure. These tablets are available through online pharmacies^30,31^, but we were not able to obtain samples for analysis.

**Figure 5:**
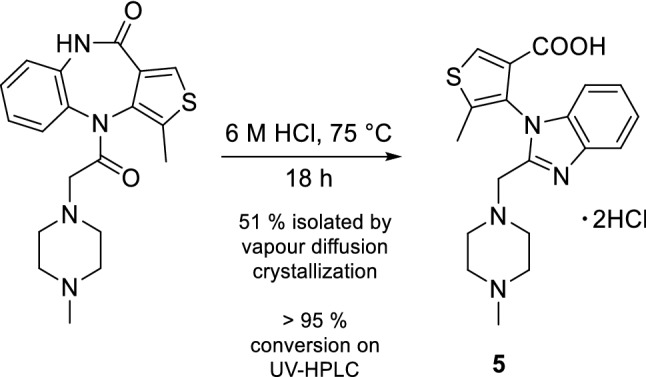
Acid catalyzed rearrangement of telenzepine.

A congener of pirenzepine, the thienobenzodiazepine telenzepine, was investigated for analog rearrangements under the same reaction conditions and we yield **5** (Fig. 5), which was characterized by NMR spectroscopy and X-ray diffraction analysis. Based on mass spectrometry data, this rearrangement was proposed previously^32^. Based on this observation, other acyl-diazepinones (*e.g.* AF-DX 116, AF-DX 384, AQ-RA 741) may be prone to this rearrangement. From a synthetic chemist’s perspective, the quantitative conversion of pirenzepine highlights the potential application of the acyl diazepinone rearrangement as a useful tool for the synthesis of other *N*-substituted imidazoles.

Although DCM is commonly regarded as inert solvent, our analysis revealed an additional curiosity of pirenzepine’s chemistry. When crystallizing pirenzepine from dichloromethane (DCM), we observed a substitution product of pirenzepine with DCM was formed (**6**, Supplementary Fig. S3, Tables S9, S10). In line with rare reports of unexpected reactions with amines^33^, it is therefore not recommended to store pirenzepine dissolved in DCM.

### Determination of affinities toward mAChR M1-M5

**Figure 6:**
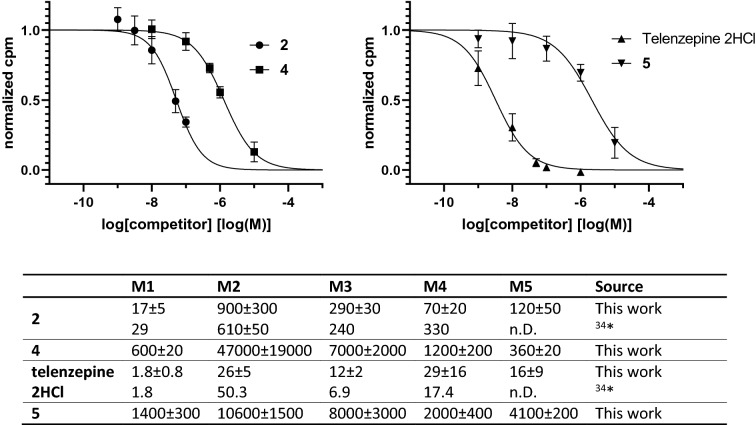
Competition binding curves using 0.2 nM [^3^H]NMS on mAChR M1 membranes for compounds **2**, **4**, **5** and telenzepine dihydrochloride. Filter bound decay rate is normalized to 1 for binding in absence of competitor and to 0 for nonspecific binding. Error bars indicate the standard deviation of n = 3 measurements. The table shows K_i_-values (nM) as determined by a competitive radioligand binding assay using [N-methyl-^3^H]scopolamine methyl chloride ([^3^H]NMS) on CHO cell membranes containing human mAChR receptors. All obtained binding displacement curves showed the expected sigmoidal shape. * Binding was carried out for 90 min at 37 °C, using membranes from rat heart tissue (M2) and transfected A9Lcells (M1 and M3) and in NG108-15 cells (M4)^34^.

The rearranged products of pirenzepine and telenzepine have significantly lower affinity towards all mAChR subtypes (Fig. 6). Regarding the affinity constants towards M1, this loss is even more pronounced in telenzepine (780-fold) compared to pirenzepine (35-fold). We tested **4** and **5** in a MTT assay for cell toxicity over the whole concentration range without measurable effect on cell viability (Supplementary Fig. S4).

### Literature research on sources of scientifically used pirenzepine

**Figure 7:**
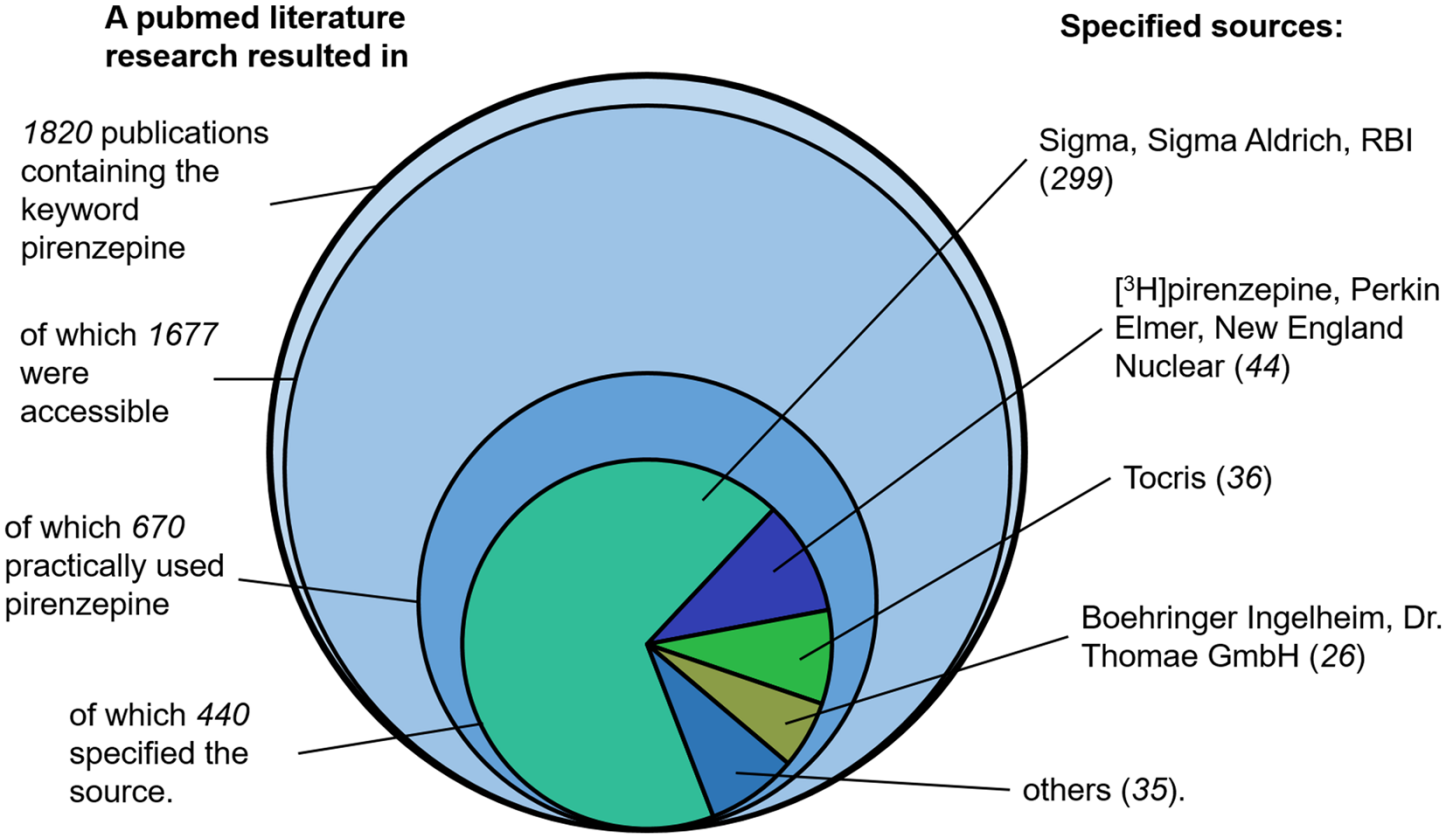
Graphical illustration of the investigated scientific literature about pirenzepine between January 2000 and February 2021 showing the literature research workflow (left) and the specified sources (right).

mAChR research relies on pirenzepine dihydrochloride to experimentally discriminate mAChR M1 from the other mAChR subtypes. Pirenzepine dihydrochloride is available as pure substance from 65 different suppliers according to SciFinder^35^. To explore the potential impact of the pirenzepine production failure on experimental reports, the pirenzepine sources in the literature since year 2000 were checked (Fig. 7).

34% of the literature which used pirenzepine did not specify the supplier. Those publications which specified a supplier never obtained the compound from an evidently affected source. In the vast majority of the investigated publications pirenzepine is used for its mAChR M1 antagonistic properties in sophisticated experimental setups. It therefore appears unlikely that the structural identity of pirenzepine would have been questioned in these experiments, given that there are numerous other factors that could cause unexpected experimental results. Therefore, the scientific literature based on pirenzepine should be reviewed carefully as any reports of unexpected findings may be due to the different chemical, biological and physiological properties of **4**.

## Conclusion

We present a case in which a chemical (**4**) not previously reported in the literature was provided instead of pirenzepine by at least one producer and two fine chemical distributors. **4** resulted from an acid catalyzed rearrangement during dihydrochloride formation. In contrast to pirenzepine, **4** showed low affinity for mAChRs. Fortunately, none of the pirenzepine-containing drugs tested within this work contained **4**. However, we were unable to test pirenzepine tablets distributed through online pharmacies provided by the affected producer.

The close structural analogue of pirenzepine, telenzepine can undergo a similar rearrangement. The product **5** suffers also from a significant loss of affinity to mAChRs. The described acyl diazepinone rearrangement may be applicable as synthetic strategy for *N*-substituted imidazoles. We strongly advise all users of pirenzepine to confirm the chemical identity of their product with the analytical methods presented within this work. Furthermore, we want to raise general awareness that fine chemicals can enter the supply chain under a wrong label and that in the case of unexpected results the declared content should be checked. We encourage a critical inspection of the certificate of analysis to avoid being a victim of chemicals sold under the wrong label. Moreover, we recommend distributors and manufactures should enter into constructive discussions with customers to improve the quality on chemical supplies.

## Experimental section

### Analytical differentiation between 2 and 4

**Pirenzepine dihydrochloride** obtained from the EDQM **(2).** ^1^H-NMR (400 MHz, D_2_O) δ 8.34 (m, 1H, Ar H-2), 7.85–7.46 (m, 6H, Ar H-3,4,7–10), 4.32 (d, 0.65H, *J* = 16.7 Hz, COCH_2_N)^Δ^, 4.23 (d, 0.35H, *J* = 16.6 Hz, COCH_2_N)*, 3.88 (d, 0.65H, *J* = 16.7 Hz, COCH_2_N)^Δ^, 3.86 (d, 0.35H, *J* = 16.6 Hz, COCH_2_N)*, 3.95–3.10 (m, 8H, H-2,3,5,6), 2.98 (s, 1.95H, CH_3_)^Δ^, 2.96 (s, 1.05H, CH_3_)*. ^13^C-NMR (100 MHz, D_2_O) δ 169.1 (NHCO)^Δ^, 168.9 (NHCO)*, 167.3 (NCO)*, 166.7 (NCO)^Δ^, 146.5 (Ar C-2) ^Δ^, 146.4 (Ar C-2)*, 144.2 (Ar C-11a)*, 143.6 (Ar C-11a)^Δ^, 139.1 (Ar C-10a)^Δ^, 138.0 (Ar C-10a)*, 135.9 (Ar C 3°)*, 135.2 (Ar C 3°)^Δ^, 133.3 (Ar C 3°)*, 133.2 (Ar C 3°)^Δ^, 132.2 (Ar C 3°)*, 131.8 (Ar C 3°)^Δ^, 131.4 (Ar C 3°)*, 131.2 (Ar C-4a)^Δ^, 130.9 (Ar C-4a)*, 130.4 (Ar C 3°)^Δ^, 129.4 (Ar C-6a)*, 128.4 (Ar C-6a)^Δ^, 128.0 (Ar C 3°)^Δ^, 127.5 (Ar C 3°)*, 127.1 (Ar C 3°)^Δ^, 126.6 (Ar C 3°)*, 57.8 (COCH_2_N)^Δ^, 57.5 (COCH_2_N)*, 51.6 (C-3,5)*, 51.5 (C-3,5)^Δ^, 50.1 (C-2,6), 43.4 (CH_3_). ^15^ N-NMR (41 MHz, D_2_O) δ 292.6 (Ar N-1), 143.7 (Ar N-11), 133.3 (Ar N-5), 40.2 (N-4). HRMS (ESI) calcd for C_19_H_22_N_5_O_2_ (M + H^+^) 352.1768, found 352.1773. m.p. 235 °C decomp.

NMR at 20 °C in D_2_O resolves the interconverting exocyclic amide bond conformations^36^. Signals marked with ‘^Δ^’ correspond to the more populated conformation, whereas ‘*’ marks signals of the less populated conformation. In case signals of both conformations coincide, no mark is given. Most tertiary aromatic carbons could not be allocated unambiguously and are therefore labelled ‘Ar C 3°’. The ^1^H signal of the amide is not visible due to the deuterium exchange from the solvent.

The compound obtained as **pirenzepine dihydrochloride** was determined to be the constitutional isomer **2-(2-((4-methylpiperazin-1-yl)methyl)-3H-imidazo[4,5-b]pyridin-3-yl)benzoic acid dihydrochloride (4).** ^1^H-NMR (400 MHz, D_2_O) δ 8.51 (dd, 1H, *J* = 5.0 Hz, *J* = 1.3 Hz, Ar H-5), 8.43 (dd, 1H, *J* = 8.3 Hz, *J* = 1.3 Hz, Ar H-7), 8.37 (dd, 1H, *J* = 7.7 Hz, *J* = 1.7 Hz, Ph H-6), 7.99 (m, 1H, Ph H-4), 7.93 (m, 1H, Ph H-5), 7.73 (m, 1H, Ph H-3), 7.72 (dd, 1H, *J* = 8.3 Hz, *J* = 5.0 Hz, Ar H-6), 4.11 (s, 2H, ArCH_2_N), 3.48 (m, 2H, H-3,5), 3.04 (m, 2H, H-3,5), 2.93 (m, 2H, H-2,6), 2.89 (s, 3H, CH_3_), 2.72 (m, 2H, H-2,6). ^13^C-NMR (100 MHz, D_2_O) δ 168.1 (COOH), 153.0 (Ar C-2), 147.2 (Ar C-5), 146.5 (Ar C-3a), 135.5 (Ph C-4), 133.3 (Ph C-6), 132.7 (Ph C-5), 131.2 (Ph C-2), 130.2 (Ph C-3), 129.1 (Ph C-1), 127.3 (Ar C-7a), 127.2 (Ar C-7), 122.7 (Ar C-6), 53.7 (C-3,5), 52.3 (ArCH_2_N), 50.3 (C-2,6), 50.0 (C-2,6), 43.4 (CH_3_). ^15^ N-NMR (41 MHz, D_2_O) δ 255.9 (Ar N-4), 180.7 (Ar N-1), 167.5 (Ar N-3), 41.6 (N-4), 36.7 (N-1). HRMS (ESI) calcd for C_19_H_22_N_5_O_2_ (M + H^+^) 352.1768, found 352.1773. m.p. 185 °C decomp.

The ^1^H signal of the carboxylic acid is not visible due to the deuterium exchange from the solvent.

### Synthesis

**5-methyl-4-(2-((4-methylpiperazin-1-yl)methyl)-1H-benzo[d]imidazol-1-yl)thiophene-3-carboxylic acid (5).** Telenzepine dihydrochloride hydrate (10 mg, 0.0226 mmol) solved in water (750 µL) and reacted with 12 M HCl (750 µL) at 75 °C for 18 h. The solvent was evaporated and the solid residue was solved in ethanol (1 mL). Crystalline **5** was obtained as colorless needles by vapour diffusion with diethyl ether (5.1 mg, 51%).

^1^H-NMR (600 MHz, D_2_O) δ 8.37 (d, 1H, *J* = 2.3 Hz, thiophene H-2), 7.92 (d, 1H, *J* = 7.1 Hz, Ar H-4), 7.68 (t, 1H, *J* = 7.1 Hz, Ar H-5), 7.62 (t, 1H, *J* = 7.1 Hz, Ar H-6), 7.43 (d, 1H, *J* = 7.1 Hz, Ar H-7), 4.04 (s, 2H, ArCH_2_N), 3.50 (brs, 2H, H-2,6), 3.11 (brs, 2H, H-2,6), 3.02 (brs, 2H, H-3,5), 2.90 (s, 3H, NCH_3_), 2.67 (brs, 2H, H-3,5), 2.30 (s, 3H, CH_3_). ^13^C-NMR (150 MHz, D_2_O) δ 164.9 (COOH), 151.2 (Ar C-2), 141.7 (thiophene C-4), 133.6 (Ar C-7a), 133.1 (thiophene C-2), 130.3 (Ar C-3a), 128.9 (thiophene C-3), 127.1 (Ar C-6), 127.0 (Ar C-5), 124.3 (thiophene C-5), 114.6 (Ar C-4), 112.4 (Ar C-7), 53.2 (C-2,6), 51.1 (ArCH_2_N), 49.5 (C-3,5), 42.7 (NCH_3_), 11.4 (CH_3_). HRMS (ESI) calcd for C_19_H_22_N_5_O_2_ (M + H^+^) 371.1536, found 371.1541.

### Kinetics

An aqueous solution of **2** (50 µL, 100 µg/mL) was combined with aqueous HCl (50 µL, 12 M) and shaken at 37 °C, 55 °C and 75 °C. 5 µL of the reaction mixture was quenched with aqueous NaHCO_3_ solution (45 µL, 55.5 mg/mL) at 30 min, 60 min, 90 min and 72 h. The experiment was repeated three times and samples analyzed by HPLC. The reaction kinetics were studied using the AUC at 216 nm of formed **4** as a function of reaction time. Values were normalized on the AUC of **4** after 72 h, which contained no detectable traces of unreacted **2**. For each temperature, data points were fitted with Y(t) = 1 − e^−kt^, with *Y* being the normalized AUC and *t* being the time. The activation energy (*E*_*A*_) was determined from linear regression based on the linearized Arrhenius equation (ln(k) =  − E_A_/(RT) + ln(A)).

### Rearrangement using HCl in [^18^O]H_2_O

**Figure 8:**
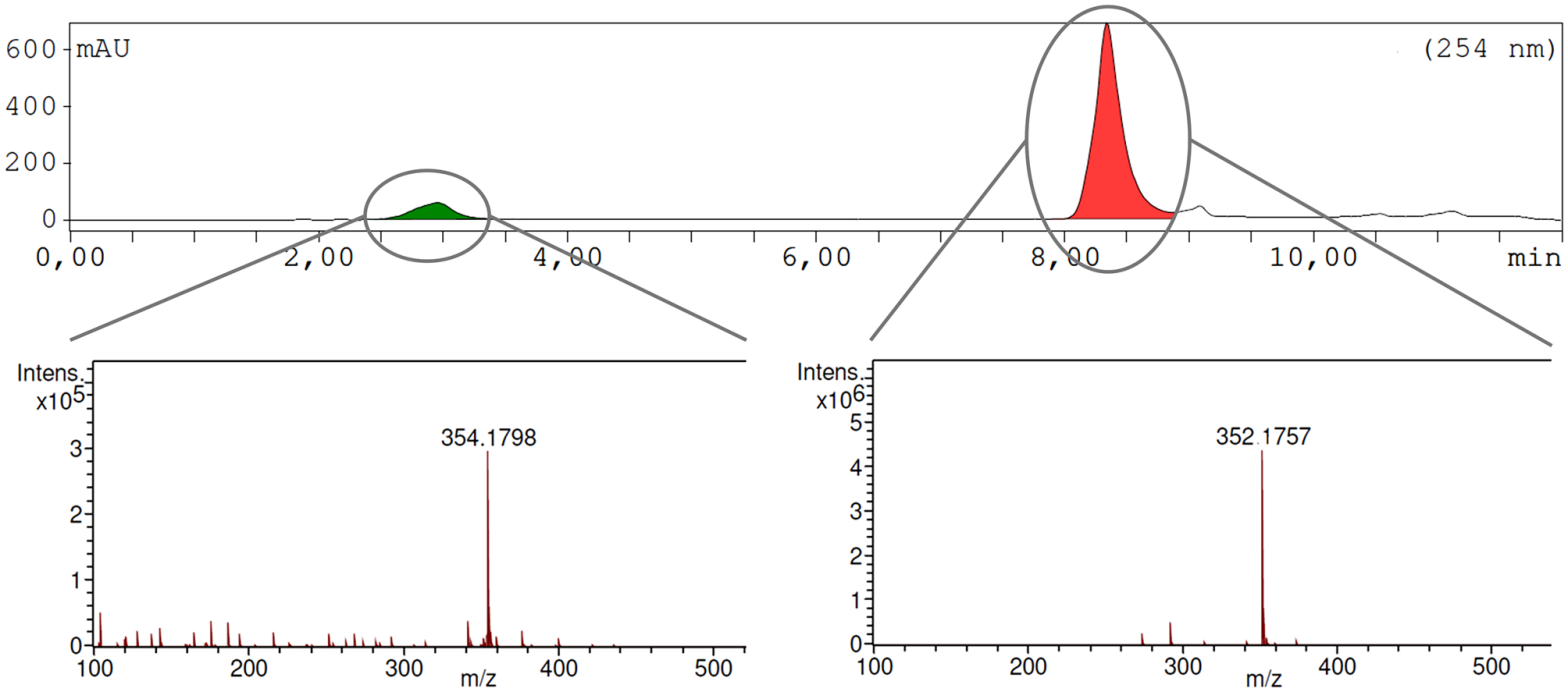
Isomerization of **2** using HCl in [^18^O]H_2_O. The UV-HPLC chromatogram and HRMS spectra of collected fractions after 60 min at 75 °C are shown: [^18^O]**4** (t_R_ = 2.9 min, expected mass: 354.1816 m/z), **2** (t_R_ = 8.3 min, expected mass: 352.1768 m/z).

Gaseous HCl was prepared from anhydrous CaCl_2_ in a three necked round-bottom flask equipped with a dropping funnel for adding concentrated aqueous HCl (10 mL). The HCl was passed through [^18^O]H_2_O (1 mL) using PVC and glass tubings. An aqueous solution of **2** in [^18^O]H_2_O (200 µL, 10 mg/mL) was combined with the prepared HCl in [^18^O]H_2_O solution (200 µL) and shaken at 75 °C for 60 min. The reaction mixture (40 µL) was quenched with aqueous NaHCO_3_ solution (100 µL, 55.5 mg/mL) and 10 µL of the resulting solution separated by HPLC. For mass spectrometry a 50 mM NH_4_Ac pH 9.3 was used as eluent instead of the (NH_4_)H_2_PO_4_ buffer. Fractions of **2** and **4** were collected and analyzed by ESI–MS (Fig. 8).

### Rearrangement in simulated gastric fluid

To mimic the intake of a 50 mg tablet of **2,** 50 µL of an aqueous solution (100 µg/mL) was combined with 2 × simulated gastric fluid (50 µL, 0.16 M aq. HCl, 2 g/L NaCl and 3.2 g/L pepsin)^37^ or equimolar aqueous HCl (50 µL) and shaken at 37 °C. The reaction mixture (5 µL) was quenched with aqueous NaHCO_3_ solution (45 µL, 55.5 mg/mL) at 30 min, 60 min, 90 min and 6 h before HPLC analysis.

### Determination of affinities toward mAChR M1-M5

Membrane preparations of mAChR M1, M2, M3, M4 or M5 receptors were produced from the respective cell line as follows. The medium of a confluent T175 cell culture flask was poured off and the cell layer was washed with ice-cold PBS. Cells were scraped off and suspended in 2 mL 10 mM Tris/HCl, 1 mM EDTA buffer (pH 7.4) and 200 µL protease inhibitor cocktail. A cell homogenate obtained from passing the cell suspension through a G29 needle was centrifuged (10 min, 1,000 g, 4 °C). The supernatant was subjected to ultracentrifugation (30 min, 100,000 g, 4 °C) and the resulting pellet was suspended in 125 µL/flask 50 mM Tris/HCl buffer (pH 7.4) and stored at -80 °C.

A competitive radioligand binding assay using [^3^H]NMS and variable concentrations of test compounds was performed in assay buffer (50 mM Tris/HCl, 10 mM MgCl_2_, 1 mM EDTA). Polypropylene tubes were filled with 5 µL aqueous solution of the test compounds, 50 µL radioligand solution in assay buffer and 445 µL membrane suspension in assay buffer (diluted approx. 1:100), shaken and incubated for 1.5 h at 20 °C. Nonspecific binding of the radioligand was determined by using 5 µL of a 1 µM scopolamine hydrobromide solution. The concentration of the radioligand solution was chosen as 2 nM, 3 nM, 8 nM, 2 nM and 10 nM for M1-M5, respectively. Membrane bound activity was recovered using a M-36 tygon tubed Cell Harvester (Brandel, Gaithersburg, MD) and GF/B filters (Whatman, GE), presoaked in 0.1% PEI for 1.5 h. After filtration of the assay mixture tubes and hosing were washed with 2 × 3 mL ice cold 50 mM Tris/HCl buffer pH 7.4. The filter paper was punched out, transferred to scintillation tubes, mixed with 2 mL Ultima Gold scintillation cocktail (high flashpoint LSC cocktail, PerkinElmer), shaken for 15 min at room temperature and counted in a liquid scintillation counter (HIDEX 300 SL) in CPM mode. Specific binding was calculated as total binding minus nonspecific binding. To delineate the IC_50_ value, specific binding was plotted against the logarithm of the test compound concentration and fitted by a variable slope logistic regression. IC_50_ values were calculated based on at least five different test compound concentrations in technical triplicates with n ≥ 3. K_i_ values were calculated by means of Cheng-Prusoff equation using K_D_ values of 0.18 nM, 0.24 nM, 0.23 nM, 0.10 nM, 0.35 nM for M1–M5, respectively^28^.

## Supplementary Information

Supplementary Information.

## Data Availability

Data of the literature research are available on request.
