# Supplementary material for: Unexpected scaffold rearrangement product of pirenzepine found in commercial samples
Source: Sci Rep. 2021 Dec 3;11:23397. doi: 10.1038/s41598-021-02732-y (PMC8642400; doi:10.1038/s41598-021-02732-y)
Supplement: Supplementary file 1 — Supplementary Information. [file 41598_2021_2732_MOESM1_ESM.pdf]

Supporting Information

Unexpected scaffold rearrangement product of pirenzepine found in commercial samples

Marius Ozenil,[1] Lukas Skos,[1] Alexander Roller,[2] Natalie Gajic,[2] Wolfgang Holzer,[3] Helmut Spreitzer,[3] Sonja Platzer-Ozenil,[4] Chrysoula Vraka,[1] Marcus Hacker,[1] Wolfgang Wadsak,[1,5] Verena Pichler[3*]

[1] Department of Biomedical Imaging and Image-guided Therapy, Division of Nuclear Medicine, Medical University of Vienna

[2] X-ray Structure Analysis Centre, Faculty of Chemistry, University of Vienna

[3] Department of Pharmaceutical Chemistry, Faculty of Life Sciences, University of Vienna

[4] Faculty of Chemistry, University of Vienna

[5] CBmed GmbH - Center for Biomarker Research in Medicine, Graz

*corresponding author

Table of contents

Table of contents 2
Materials and instrumentation 2
2D NMR and IR spectroscopy of **4** 4
Crystallographic characterization of **4** 5
Synthesis 6
Cytotoxicity toward CHO Cell Line 8
References 8

Materials and instrumentation

11-(Chloroacetyl)-5,11-dihydro-6*H*-pyrido[2,3-*b*][1,4]benzodiazepin-6-one (abcr), 1-methylpiperazine (alfa aesar), Telenzepine dihydrochloride (Sigma Aldrich, T122), scopolamine hydrobromide (Sigma Aldrich), $[^{18}O]H_2O$ (98 atom %, Hyox-18, Rotem), hydrochloric acid (HCl, 37%, Merck), pepsin (from porcine gastric mucosa, 3200-4500 units/mg protein, Sigma Aldrich), [*N*-methyl-^{3}H]scopolamine methyl chloride ([^{3}H]NMS, 2.964 TBq/µmol, 37 MBq, in 1 mL ethanol), poly(ethyleneimine) (PEI, 50% in H_2O, Fluka), phosphate buffered saline (PBS, pH 7.4, Gibco, Life Technologies Limited), acetonitrile (ACN, for HPLC, ≥99.9%, Sigma Aldrich),dichloromethane (DCM, EMPROVE® ESSENTIAL, Merck), dimethylsulfoxide (DMSO, ≥99.9%, Sigma Aldrich) and methanol (MeOH, for HPLC, ≥99.9%, Sigmal Aldrich) were used without further purification.

Table S1: *Obtained samples of fine chemical batches.*

#	company name	ordering number	lot number or batch
1	abcam	ab120153	APN10196-3-4
2	abcr	AB349569	1313238
3	AK Scientific	O383	080223G
4	EDQM Ref standard	Y0000038	batch 1
5	Glentham Life Sciences	GP0823	658NXR
6	Henan Allgreen Chemical	not reported	not reported
7	Hycultec	HY-17037	25525
8	Sigma Aldrich	P7412	079K1733V
9	TCI	P2457	XLZNG-SG

Table S2: *Obtained pharmaceuticals.*

company name	trade name	manufacturing	lot number or batch
Boehringer Ingelheim	Gastrozepin 50 mg, 50 Tabletten	Boehringer Ingelheim Ellas S.A., Greece	844033
Boehringer Ingelheim	Гастроцепін таблетки 25 мг, 50 таблеток	Boehringer Ingelheim Ellas S.A., Greece	744487
Boehringer Ingelheim	ガストロゼピン錠 100錠（10錠×10）	Nippon Boehringer Ingelheim, Japan	889001
Hikma	Gastrozepin 50 mg, 100 Tabletten	Delpharm Brétigny, France	G001
日医工株式会社 (Nichi-Iko)	ピレンゼピン塩酸塩錠25mg 100錠（10錠×10·PTP）	not specified in leaflet	CC2001
エスエス製業 (SSP Co. Ltd)	ガストール	SSP Pharmaceutical Co., Japan	01037E

NMR samples were measured in $CDCl_3$ (≥ 99.8%, stabilized with silver foil, Sigma Aldrich) or D_2O (99.9%, Sigma Aldrich) at 25 °C. Chemical shifts are given referenced to the solvent signal of ^1H in $CDCl_3$ (δ 7.26 ppm), ^1H in D_2O (δ 4.79 ppm) and ^{13}C (δ 77.0 ppm) in $CDCl_3$. ^{15}N signals are given referenced to liquid NH_3. Combination of standard NMR techniques, such as COSY, NOESY, APT, HSQC and HMBC, allowed for unambiguous assignment of NMR signals, unless stated otherwise. NMR experiments were performed with a Bruker Avance III 400 or Bruker Avance III 600 spectrometer. ESI-TOF HRMS spectra were recorded with a Bruker maXis 4G instrument. IR spectra were recorded on a Shimadzu IRAffinity-1S FTIR spectrometer. Melting points were determined at a 2°C/min ramp with a DigiMelt SRS MPA160. MMFF94 energy was determined using LigandScout 4.4.3.

HPLC analyses were performed using a XSelect HSS T3 column (3.5 µm, 100 x 4.6 mm) on an Agilent 1200 series quaternary pump and degasser equipped with an Agilent 1100 series autosampler and UV detector (upgraded to G1315B DAD). Injection volume was set to 20 µL and UV detection wavelength to 216 nm. Linear gradient programs using 25 mM $(NH_4)H_2PO_4$ pH 9.3 buffer, acetonitrile (ACN) and an initial 3 min equilibration phase were applied for the analysis of **2**, **4**, telenzepine and **5** (Table S3). For qualitative analysis of commercial pirenzepine samples the samples were injected at a concentration of 100 µg/mL. Pirenzepine containing tablets were suspended in water to yield the same concentration and insoluble additives were removed by centrifugation and/or filtration prior HPLC analysis.

Table S3: *HPLC parameters of the methods for the analysis of pirenzepine, telenzepine and their rearrangement products. LOD = limit of detection, LOL = limit of linearity*[i]

compound	t_R [min]	LOD [µg/mL]	LOL [µg/mL]	gradient
**2**	6.7	0.30	>50	0 min: 15% ACN, 3 min: 15% ACN,
**4**	3.0	0.41	>100	9 min: 60% ACN, 9.01 min: 15% ACN.
Telenzepine	7.3	0.74	>50	0 min: 12% ACN, 8 min: 45% ACN,
**5**	5.1	0.38	>50	8.25 min: 10% ACN.

Stably transfected CHO-K1 cells containing human muscarinic receptors M1-M5 were purchased from Missouri University of Science and Technology cDNA Resource Center (Cell Catalog#: CEM1000000, CEM2000000, CEM3000000, CEM4000000, CEM5000000) and maintained in Ham's F12 Nutrient Mixture (Gibco, Life Technologies Limited) supplemented with 10% FBS (Gibco, Life Technologies Limited) and 250 µg/mL Geneticin® (G418, Thermo Fisher) at 37 °C and 5% CO_2 in a cell incubator. Cells were passaged using Gibco Trypsin-EDTA (0.05%). A solution of P2714-1BTL, Sigma-Aldrich in 10 mL water was used as protease inhibitor cocktail. Count rate of the filter pieces was measured using 2 mL Ultima Gold (high flashpoint LSC cocktail, PerkinElmer) in CPM mode on a 300 SL Automatic TDCR liquid scintillation counter (HIDEX).

2D NMR and IR spectroscopy of 4

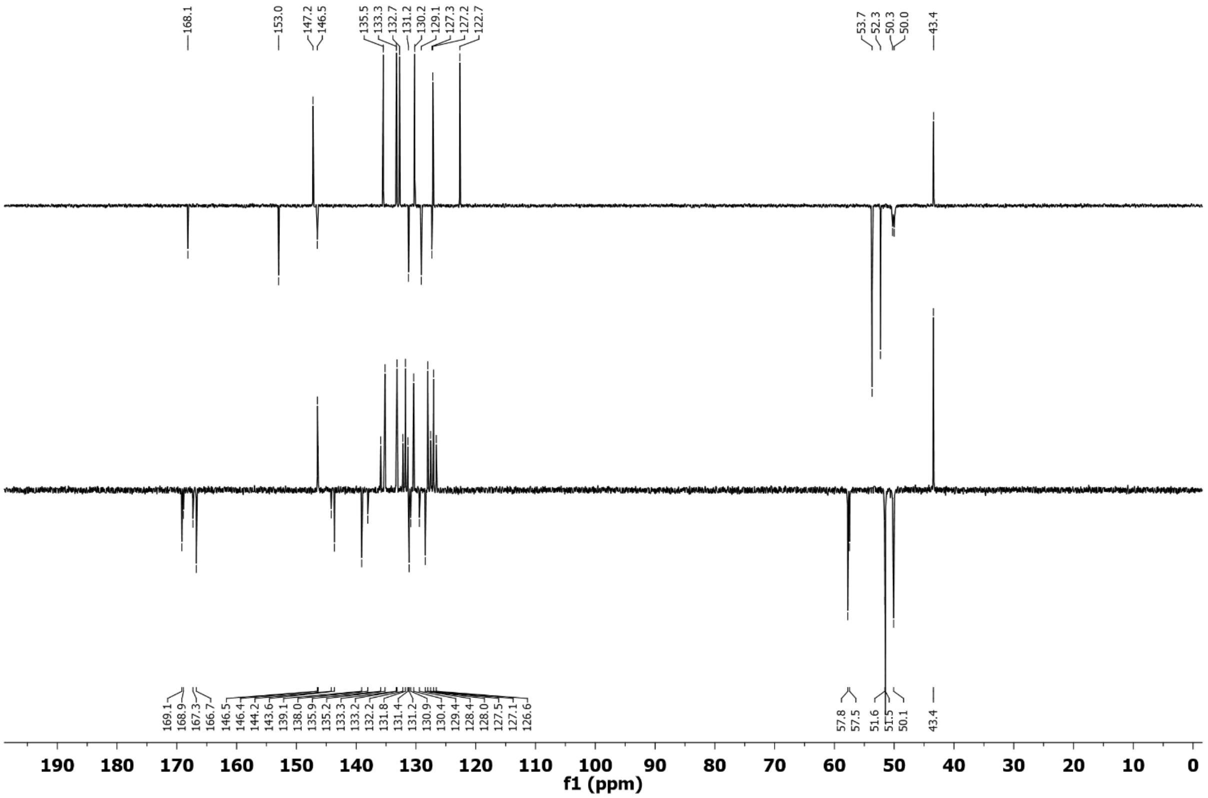

Figure S1: *^{13}C-NMR spectra of **4** (top, sample #2) and **2** (bottom, sample #4) in D_2O at 100 MHz.*

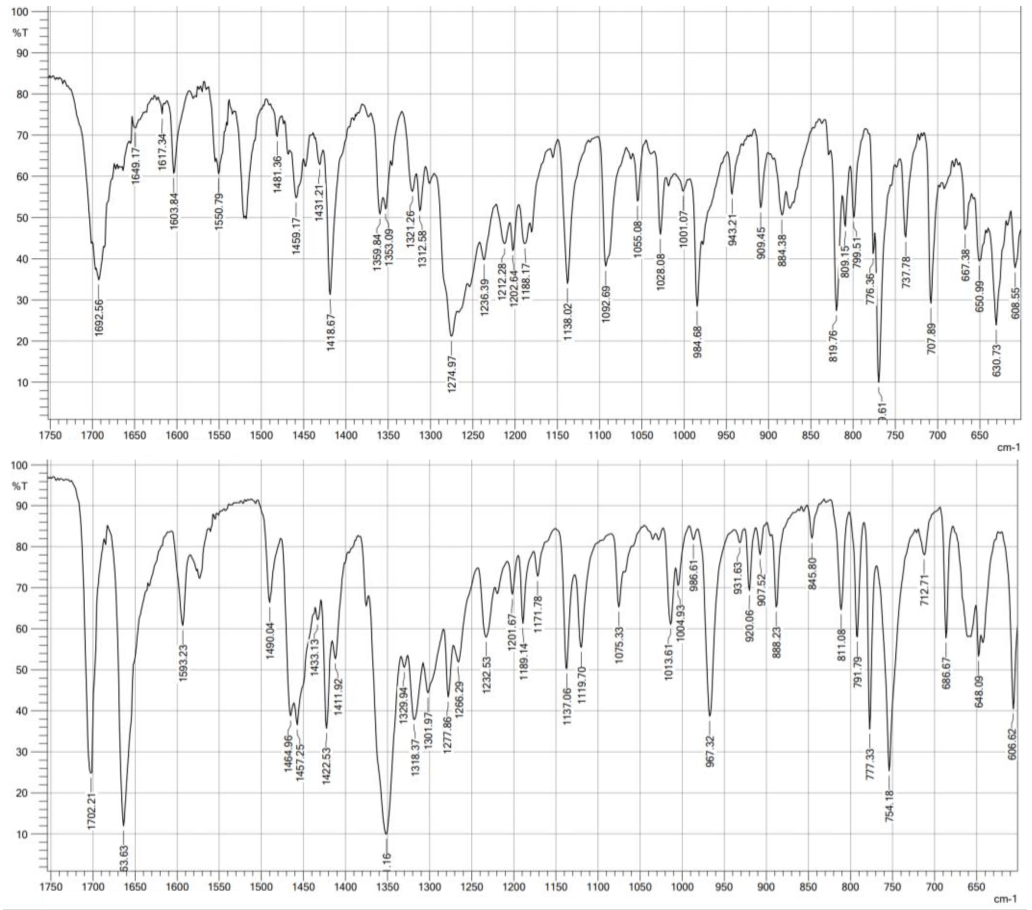

Figure S2: *IR spectra of **4** (top, sample #2) and **2** (bottom, sample #4). Most notable differences are the strong absorptions at 1663 cm^{-1} (**2**), 1351 cm^{-1} (**2**), 1275 cm^{-1} (**4**) and 1093 cm^{-1} (**4**).*

Crystallographic characterization of 4

The X-ray intensity data were measured on Bruker D8 Venture diffractometer equipped with multilayer monochromator, Mo K/α INCOATEC micro focus sealed tube and Oxford cooling system. The structures were solved by *Direct Methods, Charge Flipping and Intrinsic Phasing*. Non-hydrogen atoms were refined with *anisotropic displacement parameters*. Hydrogen atoms were inserted at calculated positions and refined with riding model. The following software was used: *Bruker SAINT software package*[ii] using a narrow-frame algorithm for frame integration, *SADABS*[iii] for absorption correction, *OLEX2*[iv] for structure solution, refinement, molecular diagrams and graphical user-interface, *Shelxle*[v] for refinement and graphical user-interface *SHELXS-2015*[vi] for structure solution, *SHELXL-2015*[vii] for refinement, *Platon*[viii] for symmetry check. Experimental data and CCDC-Codes Experimental data (Available online: http://www.ccdc.cam.ac.uk/conts/retrieving.html) can be found in Table S4. Crystal data, data collection parameters, and structure refinement details are given in Table S5 to Table S8. Asymmetric Units are visualized in Figure S3 and Figure 2.

Table S4: *Experimental parameter and CCDC-Code.*

Sample	Machine Bruker	Source	Temp. [K]	Detector Distance [mm]	Time/ Frame [s]	#Frames	Frame width [°]	CCDC
**4**	D8	Mo	100	35	8	612	0.600	2010631
**5**	D8	Mo	100	40	10	1040	0.500	2010633
**6**	D8	Mo	100	35	8	3808	0.700	2010632

Table S5: *Sample and crystal data of **4**.*

Radiation [Å]	MoKα (λ = 0.71073)	Z	4	Measurement method	\f and \w scans
Crystal habit	clear colourless block	a [Å]	9.5381(5)		
Crystal size [mm³]	0.25 × 0.22 × 0.2	b [Å]	16.2994(9)	Abs. correction type	multiscan
Empirical formula	C19H25Cl2N5O3	c [Å]	14.1925(8)	Abs. correction Tmin	
Formula weight [g/mol]	442.34	α [°]	90	Abs. correction Tmax	
Temperature [K]	100.0	β [°]	106.5352	Density (calculated) [g/cm³]	1.389
Crystal system	monoclinic	γ [°]	90	Absorption coefficient [mm⁻¹]	0.338
Space group	P21/c	Volume [Å³]	2115.2(2)	F (000) [e⁻]	928.0

Table S6: *Data collection and structure refinement of **4**.*

2Θ range for data collection [°]	4.454 to 60.33	Index ranges		Goodness-of-fit on F^2	1.034
Reflections collected	20483	h	-12 ≤ h ≤ 13	Diff. peak and hole [e⁻Å⁻³]	0.45/-0.22
Data / restraints / parameters	6239/0/280	k	-23 ≤ k ≤ 17		
Refinement method	Direct Methods	l	-20 ≤ l ≤ 19	Function minimized	$\Sigma w (F_o^2 - F_c^2)^2$
		all data	R1 = 0.0415, wR2 = 0.0873	Weighting scheme	where
		I>2σ(I)	R1 = 0.0331, wR2 = 0.0820	$w=1/[\sigma^2(Fo^2) + (0.0403P)^2 +0.7619P]$	$P=(F_o^2+2F_c^2)/3$

Table S7: *Sample and crystal data of* ***5****.*

Radiation [Å]	MoKα (λ = 0.71073)	Z	4	Measurement method	\f and \w scans
Crystal habit	clear colourless plate	a [Å]	12.8483(5)		
Crystal size [mm^3]	0.2 × 0.13 × 0.03	b [Å]	10.8713(5)	Abs. correction type	multiscan
Empirical formula	C19.75H28.51Cl2N4O3.5S	c [Å]	17.7454(7)	Abs. correction Tmin	0.5166
Formula weight [g/mol]	480.97	α [°]	90	Abs. correction Tmax	0.5619
Temperature [K]	100.0	β [°]	101.326(2)	Density (calculated) [g/cm^3]	1.314
Crystal system	monoclinic	γ [°]	90	Absorption coefficient [mm^{-1}]	0.383
Space group	P21/c	Volume [Å^3]	2430.37(18)	F (000) [e$^-$]	1012.0

Table S8: *Data collection and structure refinement of* ***5****.*

2Θ range for data collection [°]	4.682 to 50.752	Index ranges		Goodness-of-fit on F^2	1.098
Reflections collected	40495	h	$-15 \leq h \leq 15$	Diff. peak and hole [e$^-$Å^{-3}]	0.89/-0.82
Data / restraints / parameters	4441/2/288	k	$-12 \leq k \leq 13$		
Refinement method	Intrinsic Phasing	l	$-20 \leq l \leq 21$	Function minimized	Σ w $(F_o^2 - F_c^2)^2$
		all data	R1 = 0.0643, wR2 = 0.1318	Weighting scheme	where
		I>2σ(I)	R1 = 0.0487, wR2 = 0.1174	w=1/[σ^2(Fo^2) + (0.0528P)^2 +3.9477P]	P=$(F_o^2+2F_c^2)/3$

Synthesis

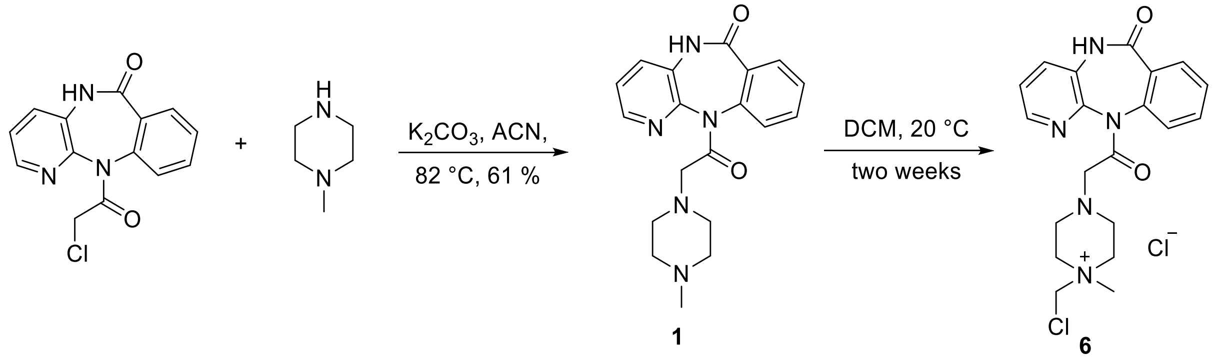

11-(2-(4-methylpiperazin-1-yl)acetyl)-5,11-dihydro-6*H*-benzo[*e*]pyrido[3,2-*b*][1,4]diazepin-6-one, trivial name: **Pirenzepine (1).** 1-Methylpiperazine (34.8 mg, 0.348 mmol) in acetonitrile (2 mL) was added dropwise to a stirred suspension of 11-(Chloroacetyl)-5,11-dihydro-6*H*-pyrido[2,3-*b*][1,4]benzodiazepin-6-one (100 mg, 0.348 mmol) and K_2CO_3 (72.1 mg, 0.521 mmol) in acetonitrile (2 mL). The suspension was refluxed for 30 min, cooled to room temperature and the solid K_2CO_3 removed by filtration. Evaporation of the filtrate afforded a residue, which was solved in dichloromethane (5 mL). 1 mL of the crude product solution was purified by preparative TLC (DCM:MeOH = 9:1, Rf = 0.19-0.32) to yield pirenzepine as a colorless oil (15 mg, 61%).

^{1}H-NMR (400MHz, $CDCl_3$) δ 9.84 (brs, 1H, CONH), 8.28 (brs, 1H, Ar H-2), 7.96 (d, *J*=7.7Hz, Ar H-7), 7.63 (m, 2H, Ar H-9,10), 7.61 (dd, *J*=7.9Hz, *J*=1.3Hz, 1H, Ar H-4), 7.42 (m, 1H, Ar H-8), 7.31 (dd, *J*=7.9Hz, *J*=4.7Hz, 1H, Ar H-3), 3.58 (d, *J*=14.6Hz, 1H, NCH_2CO), 3.19 (d, *J*=14.6Hz, 1H, NCH_2CO), 2.43 (m, 2H, H-2,6), 2.16 (s, 3H, CH_3) 2.27-2.00 (m, 6H, H-2,3,5,6). ^{13}C-NMR (100MHz, $CDCl_3$) δ 169.7 (NCO), 168.2 (NHCO), 147.4 (Ar C-11a), 144.8 (Ar C-2), 140.8 (Ar C-10a), 133.4 (Ar C-9), 131.1 (Ar C-7), 130.7 (Ar C-4a), 129.8 (Ar C-4), 128.8 (Ar C-10), 128.3 (Ar C-6a), 128.0 (Ar C-8), 123.9 (Ar C-3), 61.1 ($COCH_2N$), 54.7 (Ar C-3,5), 52.8 (Ar C-2,6), 45.9 (CH_3). ^{15}N-NMR (41MHz, $CDCl_3$) δ 301.5 (Ar N-1), 142.8 (Ar N-11), 132.5 (Ar N-5), 37.0 (N-4). HRMS (ESI) calcd for $C_{19}H_{22}N_5O_2$ (M + H$^+$) 352.1768, found 352.1776.

1-(chloromethyl)-1-methyl-4-(2-oxo-2-(6-oxo-5,6-dihydro-11*H*-benzo[*e*]pyrido[3,2-*b*][1,4]diazepin-11-yl)ethyl)piperazin-1-ium chloride (6). Upon leaving the remaining crude product solution standing for 2 weeks colorless crystals formed, which were crystallographically identified as the nucleophilic substitution product of pirenzepine with dichloromethane.

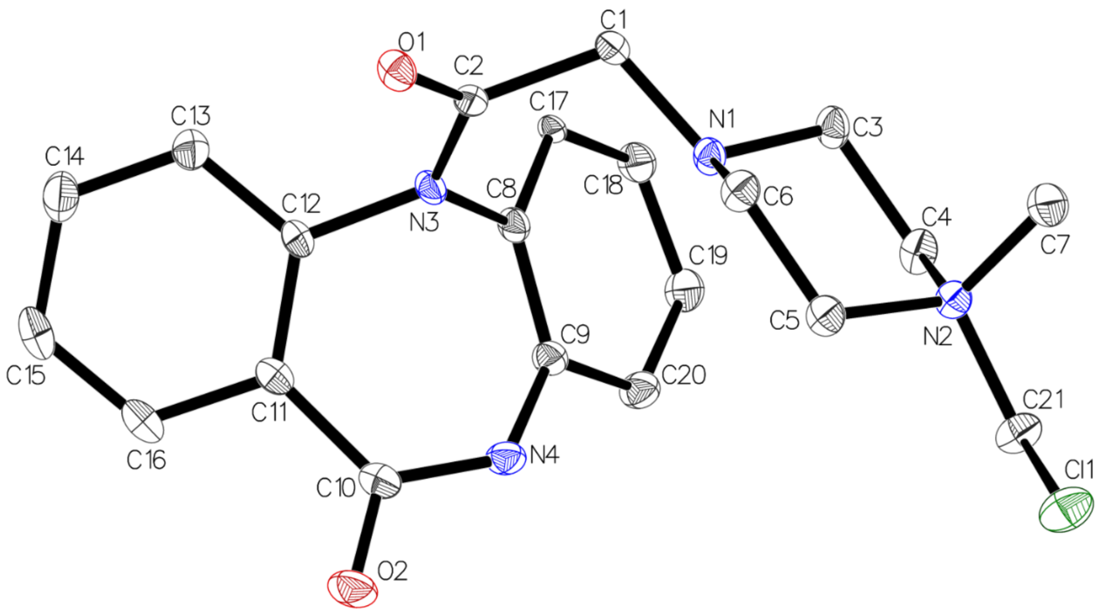

Figure S3: *Crystal structure of **6**, drawn with 50% displacement ellipsoid. The bond precision for C-C single bonds is 0.0020Å. Counter ion, hydrogen atoms and solvent omitted for clarity.*

Table S9: *Sample and crystal data of **6**.*

Radiation [Å]	MoKα (λ = 0.71073)	Z	2	Measurement method	\f and \w scans
Crystal habit	clear colourless block	a [Å]	8.9968(4)		
Crystal size [mm^3]	0.25 × 0.2 × 0.2	b [Å]	9.6557(4)	Abs. correction type	multiscan
Empirical formula	C11H13Cl2N2O	c [Å]	13.6354	Abs. correction Tmin	0.7617
Formula weight [g/mol]	260.13	α [°]	80.9712(18)	Abs. correction Tmax	0.8017
Temperature [K]	100.0	β [°]	82.0177(18)	Density (calculated) [g/cm^3]	1.491
Crystal system	triclinic	γ [°]	88.8009(19)	Absorption coefficient [mm^{-1}]	0.539
Space group	P-1	Volume [Å^3]	1158.50(8)	F (000) [e$^-$]	540.0

Table S10: *Data collection and structure refinement of **6**.*

2Θ range for data collection [°]	3.054 to 60.706	Index ranges		Goodness-of-fit on F^2	1.037
Reflections collected	74993	h	-12 ≤ h ≤ 12	Diff. peak and hole [e$^-$Å^{-3}]	1.19/-1.08
Data / restraints / parameters	6929/0/290	k	-13 ≤ k ≤ 13		
Refinement method	Charge Flipping	l	-19 ≤ l ≤ 19	Function minimized	Σ w (F_o^2 - F_c^2)^2
		all data	R1 = 0.0428, wR2 = 0.1185	Weighting scheme	where
		I>2σ(I)	R1 = 0.0388, wR2 = 0.1147	w=1/[σ^2(Fo^2) + (0.0668P)^2 +1.0659P]	P=(F_o^2+2F_c^2)/3

Cytotoxicity toward CHO Cell Line

Cytotoxicity was determined by means of a colorimetric microculture assay (MTT assay). For this purpose, CHO-K1 cells were harvested from culture flasks by trypsinization and seeded into 96-well microculture plates (Corning) in densities of 4,000 viable cells/well. After a 24 h preincubation, cells were exposed to dilutions of the test compounds in complete culture medium (200 µL/well) for 72 h. At the end of the exposure period, the compound solutions were replaced with RPMI 1640 medium supplemented with 10% heat-inactivated FBS and 4 mM L-glutamine (100 µL/well) and MTT solution (MTT reagent in PBS, 20 µL/well, 5 mg/mL). After incubation for 4 h, the medium was removed, and the formazan product was solved in DMSO (150 µL/well). Optical densities at 490 nm were measured with a microplate reader (BioTek Synergy HTX multi-mode reader) using a reference wavelength of 690 nm to correct for unspecific absorption. The quantity of viable cells was expressed in terms of T/C values by comparison to untreated controls. Evaluations were based on means from at least three independent experiments, each comprising triplicates for each concentration level.

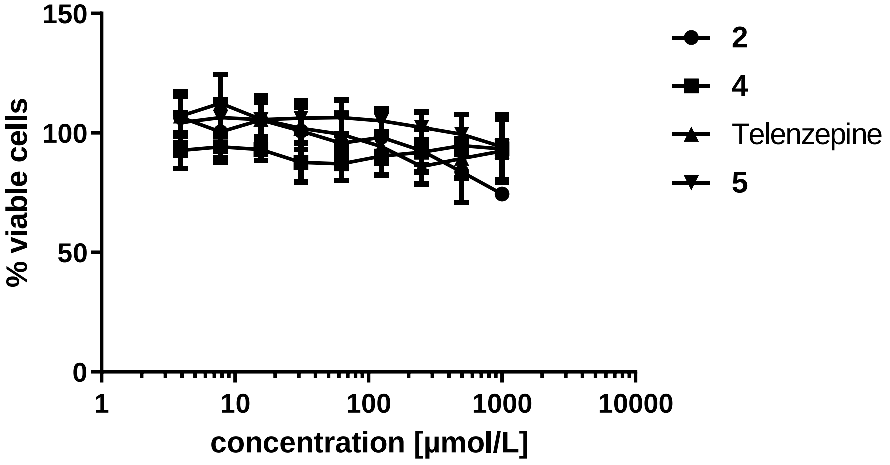

Figure S4: *Log-dose of **2**, **4**, telenzepine and **5** versus cell viability. No cytotoxic effect could be observed for concentrations as high as 1 mM.*

References

[i] ICH Harmonised Tripartite Guideline: Validation of Analytical Procedures: Text and Methodology Q2(R1), **1996**.
[ii] Bruker SAINT v8.38B Copyright © 2005-2019 Bruker AXS
[iii] G. M. Sheldrick, SADABS. University of Göttingen, Germany, **1996**.
[iv] O.V. Dolomanov, L.J. Bourhis, R.J. Gildea, J.A.K. Howard, H. Puschmann, *J. Appl. Cryst.* **2009**, *42*, 339–341.
[v] C. B. Huebschle, G. M. Sheldrick, B. Dittrich, *J. Appl. Cryst.* **2011**, *44*, 1281–1284.
[vi] G. M. Sheldrick, SHELXS v 2016/4, University of Göttingen, Germany, **2015**.
[vii] G. M. Sheldrick, SHELXL v 2016/4, University of Göttingen, Germany, **2015**.
[viii] A. L. Spek, *Acta Cryst.* **2009**, *D65*, 148–155.
